# National Institute of Mental Health Life Chart Method – Self/Prospective (NIMH-LCM-S/P™): translation and adaptation to Brazilian Portuguese

**DOI:** 10.47626/2237-6089-2020-0140

**Published:** 2021-11-09

**Authors:** Dalton Breno Costa, Luana Müller, Tatiana Quarti Irigaray, Gabriela Peretti Wagner

**Affiliations:** 1 Departamento de Psicologia Universidade Federal de Ciências da Saúde de Porto Alegre Porto Alegre RS Brazil Departamento de Psicologia, Universidade Federal de Ciências da Saúde de Porto Alegre (UFCSPA), Porto Alegre, RS, Brazil.; 2 Escola de Ciências da Saúde e da Vida Pontifícia Universidade Católica do Rio Grande do Sul Porto Alegre RS Brazil Escola de Ciências da Saúde e da Vida, Pontifícia Universidade Católica do Rio Grande do Sul (PUCRS), Porto Alegre, RS, Brazil.; 3 Escola Politécnica PUCRS Porto Alegre RS Brazil Escola Politécnica, PUCRS, Porto Alegre, RS, Brazil.

**Keywords:** Affective states, self-monitoring, mood disorders, mood

## Abstract

**Objective:**

The objective of this study was to translate and adapt the National Institute of Mental Health Life Chart Method – Self/Prospective (NIMH-LCM-S/P™) instrument for self-monitoring of mood into Brazilian Portuguese and provide evidence of content validity. Additionally, a user guide was prepared for the instrument and evaluated by mental health professionals.

**Methods:**

The study was divided into two stages – Stage 1: Translation and cross-cultural adaptation and Stage 2: Determination of content validity index (CVI) scores. The translation and cross-cultural adaptation process involved 37 participants between translators, experts, target population, and evaluators.

**Results:**

The CVI was evaluated by 15 mental health professionals. 11 (78.57%) of the items evaluated attained the maximum CVI score of 1.00, which constitutes the highest level of content validity, and no changes were suggested by participants. Only one of the items evaluated had a CVI score lower than 0.80.

**Conclusion:**

The final translated and adapted version of the NIMH-LCM-S/P™ and its user guide were evaluated by the target population and the mental health professionals. Both groups displayed satisfactory comprehension levels, suggesting there is potential for using this instrument in clinical practice to assess therapeutic interventions in Brazilian settings.

## Introduction

Mood disorders are significant and persistent mood alterations that can affect the individual’s global functioning. These disorders manifest through symptoms of depression (with or without associated anxiety) or symptoms of mania/hypomania (enhanced energy levels).^1^ The global prevalence of mood disorders is 9.6%, with women being more susceptible (4% vs. 7.3%).^2^ It is estimated that the prevalence of depression in Brazil is 5.8% among the general population.^3^

Preliminary diagnosis of mood disorders can be complex and difficult to make for many healthcare professionals.^1^ Assessment of these disorders requires a systematic and longitudinal view of many variables that can influence them. In clinical practice, changes in the mood are assessed with questionnaires and clinical observation. More traditional instruments used to assess symptoms of depression or mania, or their absence (euthymia), include the Young Mania Rating Scale (YMRS),^4^ and the Hamilton Depression Rating Scale (HDRS).^5,6^

Another way to assess changes in mood is through tracking and monitoring of said changes, which allows patients and practitioners to detect alterations and evaluate treatment effectiveness. Furthermore, this approach makes it possible to estimate future episodes of illness and prevent relapses.^7-9^ Mood can be monitored and tracked using graphs, such as life charts (a tracking method used to register the patient’s mood daily, based on their daily symptoms). Life charts make it possible to visualize mood changes along a longitudinal timeline.^9,10^

Mood monitoring based on observational research was first described by Kraepelin in 1921.^11^ Kraepelin sought to systematically and thoroughly depict his practice by recording the severity, the duration, and the characteristics of manic and depressive episodes for each patient. Over the years, researchers developed other methods to systematically assess changes in mood, such as the Systematic Treatment Enhancement Program for Bipolar Disorder (STEP-BD),^12^ the ChronoSheet,^13^ and the National Institute of Mental Health Life Chart Method (NIMH-LCM™).^14^

The NIMH-LCM™ instrument is based on Kraepelin’s model^11^ and stands out for being adaptable to different contexts and for allowing inclusion of information from different sources.^15^ The Life Chart Method (LCM™) is a public-domain instrument that was developed during the 1980s in the United States of America by the National Institute of Mental Health (NIMH).^16,17^ There are four versions of the LCM™: Life Chart Method – Clinical/ Prospective (LCM-C/P™), Life Chart Method – Self/Prospective (LCM-S/P™), Life Chart Method – Clinical/Retrospective (LCM-C/R™), and Life Chart Method – Self/Retrospective (LCM-S/R™). The differences between them are related to how the course of illness is charted (prospective or retrospective), the source of information (clinical or patient), and the levels of severity (the retrospective model has three levels and the prospective model has four levels).

With the LCM-S/P™, patients themselves can evaluate the course of illness up to four levels of episode severity. The assessment is based on classic mood disorder symptoms and the severity of functional impairment during depressive and hypomanic/manic episodes. The LCM-S/P™ also allows patients to include their medication, life events and their impact, hours of sleep, menstrual periods, symptoms of dysphoric mania, and comorbid symptoms.^14,18^

The information is charted by the patient, which allows better control of symptoms from mood disorders and faster interventions, as well as long-term recording of illness management. It can also benefit therapeutic relationships. Patients that have used the LCM™ showed an increase in euthymic periods and a decrease in the deficit (subsyndromal) of depressive and manic and hypomanic days.^19^ The LCM™ is still commonly used in clinical trials and scientific research because it assesses mood in a simple, practical, and detailed manner.^20-22^

The LCM™ instrument presented external validity when compared to other classic instruments of mood monitoring, such as the Inventory of Depressive Symptomatology-clinician-rated (IDS-C) (*r* = -0.718; *p* < 0.001), and the YMRS (*r* = 0.491; *p* = 0.001).^20^ The instrument has been also translated and adapted into German.^23^ No studies were found that administered the LCM™ in Brazil, despite the retrospective model in its clinical version (NIMH-LCM-R/C™) already having been translated and adapted to Brazilian Portuguese by Brietzke, Daruy Filho, and Grassi-Oliveira.^15^

Only a few Brazilian studies that used graphical and systematic mood monitoring were found in the literature.^24,25^ These studies mostly used mood monitoring only as a complement to assess intervention efficacy in patients with mood disorders. Moreover, they used graphs freely, with no reference, criteria, or clear and defined standards.

Mood monitoring is a comprehensive method involving large quantities of information. It is recommended that practitioners use standardized tools so that they can describe how the tools were used, the reasons for using them, and how data was analyzed.^21^ Therefore, the objective of the present study was to translate and adapt the National Institute of Mental Health Life Chart Method – Self/Prospective (NIMH-LCM-S/P™) instrument into Brazilian Portuguese, and to provide evidence of content validity. Additionally, a user guide with instructions for the NIMH-LCM-S/P™ was created and evaluated by mental health professionals.

## Methods

In this study, a combination of methods were adopted for translation and cross-cultural adaptation of the NIMH-LCM-S/P™. These methods were suggested by Borsa et al.^26^ Cassepp-Borges et al.,^27^ Gjersing et al.,^28^ Hungerbünler & Wang,^29^ and Paiano et al.^30^ According to Borsa et al.,^26^ there is no agreement on which methodological framework cross-cultural adaptation of an instrument should follow. The adaptation depends on the characteristics of each instrument, application contexts, and target population traits.

**Figure 1:**
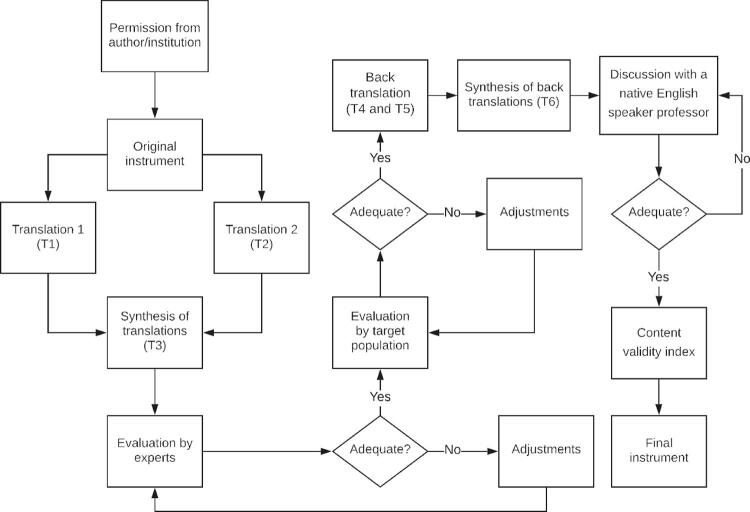
Diagram illustrating the process of translation and cross-cultural adaptation of the NIMH-LCM-S/P™. Adapted from Borsa et al.,26 Cassepp-Borges et al.,27 Gjersing et al.,28 Hungerbünler & Wang,29 and Paiano et al.30

The procedures for this investigation were divided into two stages: Stage 1 – Translation and cross-cultural adaptation; and Stage 2 – Determination of content validity index (CVI) scores. Figure 1 presents a diagram illustrating the methodological approach.

### Stage 1 – Translation and cross-cultural adaptation

Initially, prior to the translation and cross-cultural adaptation process proper, the National Institute of Mental Health Life (NIMH) was contacted in order to obtain authorization to translate and adapt the instrument to Brazilian Portuguese. The institution declared that the NIMH-LCM-S/P™ instrument is in the public domain and can therefore be adapted for other countries. Two independent translators (T1 and T2) then prepared two independent translations of the original instrument. Both translators were fluent in Brazilian Portuguese and had a high level of fluency in English and technical knowledge of mood disorders. These translations were from English to Brazilian Portuguese. A third independent translator (T3) and a committee of professionals qualified in the area then prepared a synthesis of these translations. Translator T3 had technical knowledge of mood disorders and English language certification. The committee was formed by five volunteer clinical psychologists with at least seven years of practice. They evaluated the translated version of the instrument.

The committee discussed all items on the NIMH-LCM-S/P™ instrument to investigate their suitability in Brazilian Portuguese. Adjustments were made by the committee and the translators reached a consensus among suggestions for alterations. This version of the instrument was then presented to five volunteer patients with affective mood disorders. These patients evaluated the instrument individually, for about an hour, after being instructed by their previously trained therapists. A large number of suggestions and adjustments to the instrument were made during this phase, so it was decided that the instrument should be evaluated a second time by five different patients. Six (60.00%) women and four (40.00%) men participated in this phase of the study. Regarding their educational level, five (50.00%) had completed high school, four (40.00%) had completed undergraduate degrees, and one (10.00%) had completed graduate studies. Regarding their diagnoses, three (30.00%) had type II bipolar disorder and seven (70.00%) had major depressive disorder. All patients were on medical treatment and in a euthymic episode.

Additional adjustments were made after the patients’ evaluation of the translation and the instrument items. Back-translations of the instrument (with the suggested alterations made) from Brazilian Portuguese to English were performed by three independent translators (T4, T5, and T6). These three translators had English language certification, extensive knowledge of Brazilian Portuguese, and technical knowledge of mood disorders. The back-translations were sent for evaluation to a Canadian professor who is a native English speaker and mental health professional, thus ensuring the validity of the translation.

### Stage 2 – Content validity index (CVI)

After the adjustments suggested during the back translation step had been made, the final version in Brazilian Portuguese was used to determine the CVI scores. Items were assessed for suitability using closed-ended questions (“yes” or “no”). A space for comments was also included for each item evaluated, because closed-ended questions can be limiting. Evaluators could thus comment on further aspects, reducing the likelihood of biases during the approval process by expert evaluators.^31^ Data were collected through an online platform called Qualtrics Survey Software.

After that, the answers were used to calculate the CVI for each item with the formula CVI = (ne - N/2)/ (N/2) (where, ne = the number of evaluators that rated an item as essential; and N = the total number of evaluators).^32^ This formula is widely used for analyses such as this.^33,34^

### Participants in the CVI stage

The CVI evaluation was performed by 15 mental health professionals – psychiatrists as well as psychologists. The number of evaluators was higher than what is recommended in the literature, which is 2 to 10 evaluators.^35^ Eleven (73.30%) were women; ten (66.67%) had a degree in Psychology, and five (33.33%) had a medical degree and residency in psychiatry. Their mean experience in the area was 10.93 years (SD = 6.60 years), with a range of 2 to 28 years of experience. Volunteers were recruited using an electronic form, which was completed by the volunteers themselves.

### Instruments

#### Form with evaluators’ (experts) information

This covered the following variables: age, gender, education (undergraduate, specialization, master’s degree, or doctorate), and years spent in education.

#### National Institute of Mental Health Life Chart Method – Self/Prospective (NIMH-LCM-S/P™)^14^,^18^

Instrument created by the NIMH. This instrument is used by patients to monitor mood daily and prospectively. The course of illness can be rated at one of four levels of episode severity. Monitoring is based on classic mood disorder symptoms and the severity of functional impairment during depressive and hypomanic/manic episodes.

This instrument also contains items for including additional information associated with mood in the monitoring, such as medication (especially the number of pills ingested), hours of sleep, menstrual periods, and symptoms of dysphoric mania. It also provides space to note life events and their impact. In other words, the respondent can report a life event and the degree of impact of this event, considering a scale that ranges from -4 to +4. An event with a -4 grade represents an extremely negative experience, a 0 grade represents a neutral experience, and a +4 grade represents an extremely positive life event.

## Data analysis procedures

Data were analyzed and organized in a database, which was created using the Statistical Package for the Social Sciences (SPSS) version 23 for Windows. Data were described as absolute and relative frequencies for qualitative variables and mean and standard deviation for quantitative variables.

## Ethical procedures

This study meets all the guidelines and regulatory standards for research involving human beings, which can be found in National Health Committee Resolutions 466/2012 and 510/2016.^36,37^ Researchers conducted all ethical care procedures to ensure anonymity in data treatment and dissemination of research findings. The research protocol was submitted for evaluation to the ethics committee at PUCRS and approved under CAAE: 05632819.5.0000.5336. Study participants were informed about the purpose of the research and given an informed consent form to sign. This covered research objectives and procedures as well as the guarantee of anonymity. Each volunteer participant kept a copy of the form they signed.

## Results

### Translation and cross-cultural adaptation

The translation and cross-cultural adaptation process involved six translators, a committee of professionals formed by five psychologists, ten patients diagnosed with affective mood disorders, and a native English speaking professor specialized in mental health. During this process, suggestions for alterations were made by the participants in each phase. The main suggestions and alterations can be seen in Table 1.

**Table 1:** The main suggestions, alterations, and procedures adopted during the process of translation and cross-cultural adaptation of the NIMH-LCM-S/PTM

Items	Phase	Suggestions	Alterations
General structure of the instrument	Evaluation by the target population	Distribution of items on additional sheets of paper, more space for notes	The original instrument was reorganized to use two pages. The life events and impact items and the list of comorbid symptoms were moved to a second page of the instrument. Font size, font type and graphs were also altered to optimize space for notes. The NIMH-LCM-S/P™ method was preserved.
Dotted lines	Evaluation by the target population	Substitution of the dotted lines in the mood severity note section for something more intuitive and easier to understand. Suggestion: circles that can be colored-in.	Suggestions were taken into consideration. The dotted lines were substituted by circles that can be colored-in.
Instructions	Evaluation by the target population and during the evaluation by professionals	Creation of a guide for patients and summarized instructions of the instrument.	Suggestions were taken into consideration. A user guide for using the instrument was created, and the summarized instructions were integrated into the instrument.

The user guide and the summarized instructions were created by the authors at the request of the participants and professionals involved. The NIMH Life Chart Manual for Recurrent Affective Illness: The LCM-S/P (Self-Version/Prospective),^18^ among other references,^38,39^ served as a basis. The authors sought to use plain language to facilitate patient understanding of the instrument. All necessary information for using the instrument was added to the guide in the form of instructions.

Given the number of adjustments during the evaluation by the target population, it was decided that the instrument should be evaluated a second time by five different patients with mood affective disorders. The original version of the instrument and the final version of the NIMH-LCM-S/P™ translation can be found in Figure S1, available as online-only supplementary material.

### Content validity index (CVI)

The CVI scores resulting from the professional analysis are shown in Table 2. It can be observed that all evaluated items obtained a CVI above the cutoff suggested by Hutz et al.^34^ for 15 evaluators. The minimum CVI suggested was 0.49.

**Table 2:** Level of agreement among evaluators in relation to items of the NIMH-LCM-S/PTM, summarized instructions, and NIMH-LCM-S/PTM user guide

Items	Frequency	%	CVI
1 - NIMH-LCM-S/P – Medication	15	100.00	1.00
2 - NIMH-LCM-S/P – Hours of Sleep	15	100.00	1.00
3 - NIMH-LCM-S/P – Severity of Mood	15	100.00	1.00
4 - NIMH-LCM-S/P – Mood Scale	15	100.00	1.00
5 - NIMH-LCM-S/P – Number of Mood Switches	14	93.33	0.86
6 - NIMH-LCM-S/P – Menstrual Cycle	14	93.33	0.86
7 - NIMH-LCM-S/P – Life Events	15	100.00	1.00
8 - NIMH-LCM-S/P – Comorbid Symptoms	15	100.00	1.00
Summarized instructions – Introduction	13	86.67	0.73
Summarized instructions – Symptoms	15	100.00	1.00
Summarized instructions – User guide	15	100.00	1.00
User guide – Presentation	15	100.00	1.00
User guide – Introduction	15	100.00	1.00
User guide – Instructions	15	100.00	1.00

CVI = content validity index.

Regarding level of comprehensibility, 11 (78.57%) of the evaluated items obtained a high CVI. No suggestions for alterations were made by the evaluators. Suggestions for alterations to other items were discussed and then implemented by the committee of experts. The alterations concerned Brazilian Portuguese spelling and grammatical agreement. Some alterations were also made to the instrument. For example, according to evaluators, the item for menstrual periods lacked clarity. It was not clear that only the days on which menstruation occurs should be marked. In this case, the committee of experts opted to keep the item in the instrument as it had been translated, while improving the instructions in the user guide. It was also suggested that the word “intensity” be changed to “severity” in item 3 of the instrument.

## Discussion

The objective of this study was to translate and adapt the National Institute of Mental Health Life Chart Method – Self/Prospective (NIMH-LCM-S/PTM) mood self-monitoring instrument for Brazilian Portuguese and to provide evidence of content validity. Additionally, a user guide on how to use and fill in the instrument was prepared and evaluated (Figure S1 and Appendix S2, available as online-only supplementary materials). At the end of the translation evaluation process, it was observed that most of the evaluated items achieved the maximum CVI. This result indicates item equivalence for the Brazilian Portuguese version, as well as a substantial agreement among evaluators in the agreement interval. One of the objectives of a rigorous translation and cross-cultural adaptation process is to guarantee equivalence regardless of the context in which the instrument is used.^26,40^

Borsa et al.^26^ stress that instruments must be evaluated by the target population to verify item suitability throughout the cross-cultural adaptation process. These authors also emphasize that this evaluation must be done one or more times if necessary. In this study, two evaluations by the target population were needed because of initial difficulties understanding the instrument. The suggestions provided were used to alter the instrument in order to make it more understandable and suitable for the target population.

Just one of the items evaluated by the expert evaluators attained a value less than 0.80 for agreement. This item referred to the introduction of the summarized instructions and suggestions were made to improve the way it was written to achieve better understanding by the target population. Concerning agreement among the judges of the NIMH-LCM-S/P™ instrument, all items achieved values equal to or greater than 0.86. The literature considers that a CVI or agreement between evaluators greater than 0.80 is excellent.^34^ None of the items evaluated attained a CVI value below 0.49, which is the minimum suggested by Hutz et al.^34^ when there are 15 evaluators. Therefore, none of the items were removed.

In this study, the instrument layout was modified based on suggestions from the target population. The original methodology was preserved. Additionally, a user guide for filling out the instrument was developed and included in the summarized instructions, which improves target population comprehension, thus following Gregoire’s recommendations^40^ that all items and instructions should be suitable for and intelligible to the target population. However, identifying mood can still be difficult for patients. Patients may still need help from a mental health professional to understand the instrument and to learn how to identify their own mood, especially patients with lower levels of education or lack of insight.

To date, there are no prospective mood-monitoring instruments in Brazilian literature with rigorous translation and adaptation methodology that can be used to monitor a patient’s mood. In regard to this, the strong reliability of this Brazilian Portuguese version of the instrument seems to fill in this gap. This result suggests that the NIMH-LCM-S/P™ instrument can be used in clinical practice to assess therapeutic interventions longitudinally, which will help patients, health professionals, and researchers to assess and better comprehend mood symptoms.^20^ It is possible, through self-monitoring, to become acquainted with mood changes to estimate future episodes of illness, response to medication, and increase in euthymic periods, as well as prevention of relapse.^7-9^ It is also possible to assess the number, sequence, intensity, and duration of each episode, among other items that can influence mood (hours of sleep, medication, menstrual cycle, life events).^9,10^

The final version of the translation and adaptation of the NIMH-LCM-S/P™ and its user guide were evaluated by the target population and mental health professionals. Both groups displayed satisfactory comprehension levels, which suggest the potential for using this instrument in clinical practice to assess therapeutic interventions in Brazilian settings. However, this study has only initiated the process of instrument evaluation. It is necessary to obtain other types of evidence of validity, such as convergent validity, by observing correlations between this instrument and other instruments that are already traditional in mood assessment. Future studies are also recommended to adapt this method to the Brazilian context in virtual environments, as already done in other countries.^41-43^ Regardless, this is an instrument that is accessible and in the public domain. It helps patients in their self-monitoring of mood using graphs and longitudinal tracking, which enables patients and practitioners to monitor patient evolution and treatment.

## Supplementary material



## References

[1] 1. Associação Americana de Psiquiatria. Manual Diagnóstico e Estatístico de Transtornos Mentais, 5ª edição (DSM-5). Porto Alegre: Artmed; 2014.

[2] 2. Steel Z, Marnane C, Iranpour C, Chey T, Jackson JW, Patel V, Silove D. The global prevalence of common mental disorders: a systematic review 45 and meta-analysis 1980-2013. Int J Epidemiol. 2014;43:476-93. 10.1093/ije/dyu038 PMC3997379 24648481

[3] 3. World Health Organization (WHO). Depression and other common mental disorders: global health estimates. Geneva: WHO; 2017.

[4] 4. Young RC, Biggs JT, Ziegler VE, Meyer DA. A rating scale for mania: reliability, validity and sensitivity. Br J Psychiatry. 1978;133:429-35. 10.1192/bjp.133.5.429 728692

[5] 5. Hamilton M. A rating scale for depression. J Neurol Neurosurg Psychiatry. 1960;23:56-62. 10.1136/jnnp.23.1.56 PMC495331 14399272

[6] 6. Swartz HA, Rucci P, Thase ME, Wallace M, Carretta E, Celedonia KL, et al. Psychotherapy alone and combined with medication as treatments for bipolar II depression: a randomized controlled trial. J Clin Psychiatry. 2018;79:16m11027. 10.4088/JCP.16m11027 PMC5823786 28703949

[7] 7. Basco MR. Vencendo o transtorno bipolar com terapia cognitivo-comportamental. tratamentos que funcionam: manual do paciente. Porto Alegre: Artmed; 2009.

[8] 8. Faurholt-Jepsen M, Munkholm K, Frost M, Bardram JE, Kessing LV. Electronic self-monitoring of mood using IT platforms in adult patients with bipolar disorder: a systematic review of the validity and evidence. BMC Psychiatry. 2016;16:1-14. 10.1186/s12888-016-0713-0 PMC4714425 26769120

[9] 9. Vieira TC, Marques EL. Possíveis estratégias e técnicas de manejo para o transtorno bipolar na perspectiva cognitivo-comportamental. Psicologia.pt. 2017;1:1-19.

[10] 10. Lotufo Neto F. [Cognitive behavioral therapy for bipolar disorders]. Braz J Psychiatry. 2004;26 Supl3:44-6. 10.1590/s1516-44462004000700010 15597139

[11] 11. Kraepelin E. Manic depressive insanity and paranoia. J Nerv Ment Dis. 1921;53:350.

[12] 12. Sachs GS, Thase ME, Otto MW, Bauer M, Miklowitz D, Wisniewski SR, et al. Rationale, design, and methods of the systematic treatment enhancement program for bipolar disorder (STEP-BD). Biol Psychiatry. 2003;53:1028-42. 10.1016/s0006-3223(03)00165-3 12788248

[13] 13. Bauer MS, Crits-Christoph P, Ball WA, Dewees E, McAllister T, Alahi P, et al. Independent assessment of manic and depressive symptoms by self-rating: scale characteristics and implications for the study of mania. Arch Gen Psychiatry. 1991;48:807-12. 10.1001/archpsyc.1991.01810330031005 1929771

[14] 14. Leverich G, Post R. Life charting of affective disorders. CNS Spectr. 1998;3:21-37.

[15] 15. Brietzke E, Daruy Filho L, Grassi-Oliveira R. “Life Chart” retrospectivo: instrumento para assinalar graficamente a presença e a evolução do impacto funcional de episódios afetivos maníacos e depressivos. Rev Psiquiatr Clin. 2009;36:217-20.

[16] 16. Post R, Roy-Byrne PP, Uhde T. Graphic representation of the life course of illness in patients with affective disorder. Am J Psychiatry. 1988;145:844-8. 10.1176/ajp.145.7.844 3381929

[17] 17. Roy-Byrne P, Post RM, Uhde TW, Porcu T, Davis D. The longitudinal course of recurrent affective illness: life chart data from research patients at the NIMH. Acta Psychiatr Scand. 1985;71:1-33. 10.1111/j.1600-0447.1985.tb10510.x 3861072

[18] 18. Leverich GS, Post RM. The NIMH life chart manual for recurrent affective illness: the LCM - S/P (self-version/prospective) [Internet]. 2002 [cited 2021 Mar 11]. www.bipolarnews.org/pdfs/Clinician%20Prospective%20Manual.pdf

[19] 19. Born C, Seitz NN, Grunze H, Vieta E, Dittmann S, Seemüller F, et al. Preliminary results of a fine-grain analysis of mood swings and treatment modalities of bipolar I and II patients using the daily prospective life-chart-methodology. Acta Psychiatr Scand. 2009;120:474-80. 10.1111/j.1600-0447.2009.01412.x 19485960

[20] 20. Born C, Amann BL, Grunze H, Post RM, Schärer L. Saving time and money: a validation of the self-ratings on the prospective NIMH Life-Chart Method (NIMH-LCM). BMC Psychiatry. 2014;14:1-7. 10.1186/1471-244X-14-130 PMC4031162 24886463

[21] 21. Koenders MA, Nolen WA, Giltay EJ, Hoencamp E, Spijker AT. The use of the prospective NIMH Life Chart Method as a bipolar mood assessment method in research: a systematic review of different methods, outcome measures and interpretations. J Affect Disord. 2015;175:260-8. 10.1016/j.jad.2015.01.005 25658502

[22] 22. Post RM, Altshuler LL, Frye MA, Suppes T, Keck JP, McElroy SL, et al. Complexity of pharmacologic treatment required for sustained improvement in outpatients with bipolar disorder. J Clin Psychiatry. 2010;71:1176-86. 10.4088/JCP.08m04811yel 20923622

[23] 23. Akkerhuis GW, Kupka RW, Honig A, Nolen WA. Handleiding Lifechart Methode voor Stemmingsstoornissen (Manual for Life-Chart Method). Utrecht: Interne Uitgave Willem Arntsz Huis; 1996.

[24] 24. Costa RS. Transtorno bipolar: contribuições de uma intervenção analítico-comportamental em grupo [dissertation]. Londrina: Universidade Estadual de Londrina; 2016.

[25] 25. Mussi SV, Soares MRZ, Grossi R. Transtorno bipolar: avaliação de um programa de psicoeducação sob o enfoque da análise do comportamento. Rev Bras Ter Comport Cogn. 2013;15:45-63.

[26] 26. Borsa JC, Damásio BF, Bandeira DR. Adaptação e validação de instrumentos psicológicos entre culturas: algumas considerações. Paidéia. 2012;22:423-32.

[27] 27. Cassepp-Borges V, Balbinotti MAA, Teodoro MLM. Tradução e validação de conteúdo: uma proposta para a adaptação de instrumentos. In: Pasquali L, colaboradores, editors. Instrumentação psicológica: fundamentos e práticas. Porto Alegre: Artmed; 2010. p. 506-20.

[28] 28. Gjersing L, Caplehorn JR, Clausen T. Cross-cultural adaptation of research instruments: language, setting, time and statistical. BMC Med Res Methodol. 2010;10:1-10. 10.1186/1471-2288-10-13 PMC2831007 20144247

[29] 29. Hungerbünler I, Wang YP. Aspectos transculturais na adaptação de instrumentos. In: Gorentein C, Wang YP, Hungerbühler Ines, editors. Instrumentos de avaliação em saúde mental. Porto Alegre: Artmed; 2016. p. 12-7.

[30] 30. Paiano R, Teixeira MCTV, Cantiere CN, Efstratopoulou MA, Carreiro LR. Translation and cross-cultural adaptation of the Motor Behavior Checklist (MBC) into Brazilian Portuguese. Trends Psychiatry Psychother.2019;41:167-75. 10.1590/2237-6089-2017-0104 31166562

[31] 31. Gisev N, Bell JS, Chen TF. Interrater agreement and interrater reliability: key concepts, approaches, and applications. Res Social Adm Pharm. 2013;9:330-8. 10.1016/j.sapharm.2012.04.004 22695215

[32] 32. Lawshe CH. A quantitative approach to content validity. Pers Psychol. 1975;28:563-75.

[33] 33. Ayre C, Scally AJ. Critical values for Lawshe’s content validity ratio: revisiting the original methods of calculation. Meas Eval Couns Dev.2014;47:79-86.

[34] 34. Hutz CS, Bandeira DR, Trentini CM. Psicometria. Porto Alegre: Artmed; 2015.

[35] 35. Damásio BF, Borsa JC. Manual de desenvolvimento de instrumentos psicológicos. São Paulo: Vetor; 2018.

[36] 36. Brasil, Ministério da saúde. Resolução nº 466, de 12 de dezembro de 2012. Diário Oficial da União, 13 junho 2013. conselho.saude.gov.br/resolucoes/2012/Reso466.pdf

[37] 37. Brasil, Ministério da saúde. Resolução nº 510, de 7 de abril de 2016. Diário Oficial da União 2016. bvsms.saude.gov.br/bvs/saudelegis/cns/2016/res0510_07_04_2016.html

[38] 38. Ekkekakis P. The measurement of affect, mood, and emotion: a guide for health-behavioral research. New York: Cambridge University; 2013.

[39] 39. Robbins SP, Judge TA. Organizational behavior. Bonston: Person; 2012.

[40] 40. Gregoire J. ITC guidelines for translating and adapting tests. Int J Test. 2018;18:101-34.

[41] 41. Schärer LO, Hartweg V, Valerius G, Graf M, Hoern M, Biedermann C, et al. Life charts on a palmtop computer: first results of a feasibility study with an electronic diary for bipolar patients. Bipolar Disord. 2002;4:107-8. 10.1034/j.1399-5618.4.s1.51.x 12479693

[42] 42. Schärer LO, Krienke UJ, Graf SM, Meltzer K, Langosch JM. Validation of life-charts documented with the personal life-chart app - a self-monitoring tool for bipolar disorder. BMC Psychiatry. 2015;15:1-7. 10.1186/s12888-015-0414-0 PMC4367878 25885225

[43] 43. Lieberman DZ, Kelly TF, Douglas L, Goodwin FK. A randomized comparison of online and paper mood charts for people with bipolar disorder. J Affect Disord. 2010;124:85-9. 10.1016/j.jad.2009.10.019 19896202

