# Supplementary material for: National Institute of Mental Health Life Chart Method – Self/Prospective (NIMH-LCM-S/P™): translation and adaptation to Brazilian Portuguese
Source: Trends Psychiatry Psychother. 2021 Nov 9;44:e20200140. doi: 10.47626/2237-6089-2020-0140 (PMC9911163; doi:10.47626/2237-6089-2020-0140)

http://dx.doi.org/10.47626/2237-6089-2020-0140


**Figure S1** - Original version of the National Institute of Mental Health Life Chart Method - Self/Prospective™

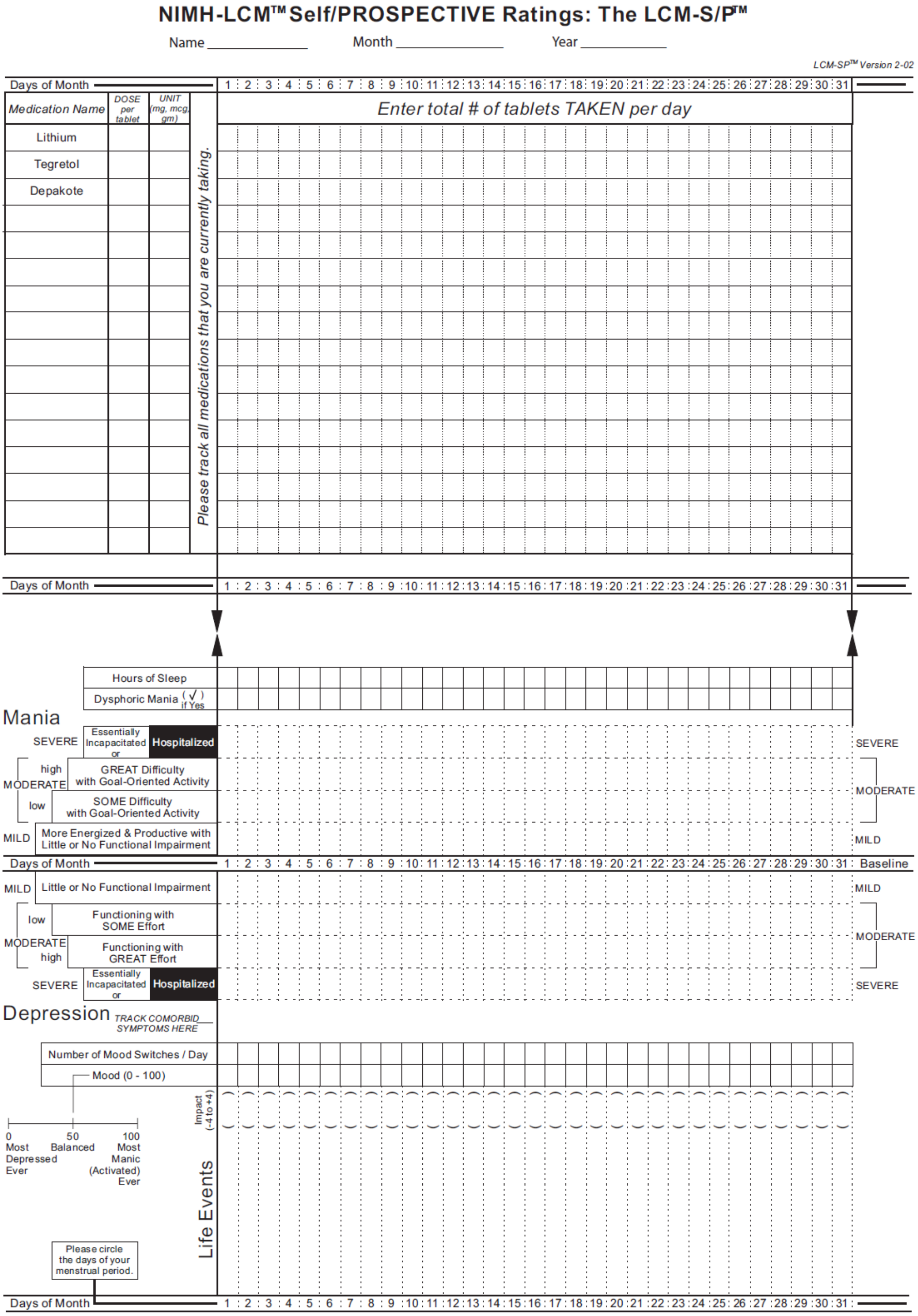

Supplement: Supplementary file 1 [file 2238-0019-trends-44-e20200140-suppl01.pdf]
