# Supplementary material for: National Institute of Mental Health Life Chart Method – Self/Prospective (NIMH-LCM-S/P™): translation and adaptation to Brazilian Portuguese
Source: Trends Psychiatry Psychother. 2021 Nov 9;44:e20200140. doi: 10.47626/2237-6089-2020-0140 (PMC9911163; doi:10.47626/2237-6089-2020-0140)
Supplement: Supplementary file 2 [file 2238-0019-trends-44-e20200140-suppl02.pdf]

http://dx.doi.org/10.47626/2237-6089-2020-0140

Appendix S2 - Brazilian version of the National Institute of Mental Health Life Chart Method - Self/Prospective™

GUIA DE PREENCHIMENTO DA VERSÃO BRASILEIRA DO *LIFE CHART METHOD - SELF/PROSPECTIVE* (LCM-S/P)

APRESENTAÇÃO

Automonitoramento do Humor com Afetivogramas

O automonitoramento do humor é uma técnica utilizada no tratamento de pacientes com oscilações de humor, pois ajuda tanto o paciente, quanto o clínico a compreenderem como ocorrem as oscilações de humor. A técnica consiste em uma autoavaliação do humor pelo paciente, seguida de anotação ou registro do humor em algum diário.

Uma das formas de anotar e acompanhar o humor é por meio da construção de gráficos, como os **afetivogramas**. Com esse modelo de anotação, é possível visualizar graficamente as alterações de humor em uma linha do tempo.

Depois de um período de monitoramento do humor realizado pelo paciente, o clínico terá um material conciso para diagnosticar de forma mais precisa o seu paciente. Além disso, poderá escolher a melhor linha de tratamento, acompanhar a evolução do paciente, estimar precocemente novos episódios de humor e prevenir recaídas.

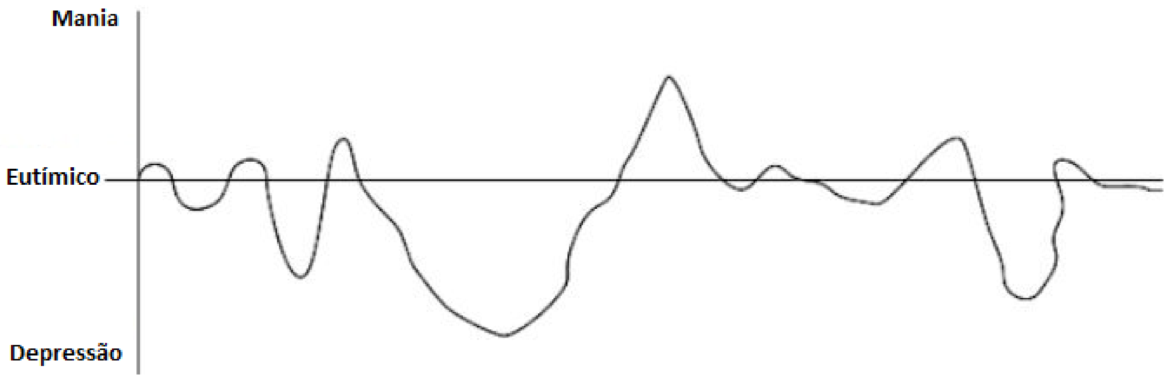

Figura 1. Exemplo de um Afetivograma. Figura desenvolvida pelos autores.

Um dos diários de anotações de humor mais utilizados é o *Life Chart Method* (LCM), desenvolvido pelo *National Institute of Mental Health* (NIMH), que se destaca pela sua flexibilidade em diferentes contextos e possibilita a inclusão de diversas outras informações. O LCM possui diversas versões e objetiva auxiliar pacientes e clínicos no controle e visualização das oscilações de humor. Neste guia, vamos trabalhar com a versão *Life Chart Method - Self/Prospective* (LCM-S/P), que é a versão prospectiva e preenchida pelo próprio paciente. A seguir, serão apresentadas as instruções e as orientações de como preencher e realizar o monitoramento de humor utilizando o *LCM-S/P*.

INTRODUÇÃO

O que é humor?

Muitas pessoas pensam que humor é a emoção ou sentimento que sentimos em determinados momentos, mas esse pensamento não é verdadeiro. Para entendermos melhor o que é humor precisamos diferenciá-lo de emoção e de afeto (Ekkekakis, 2013; Robbins & Judge, 2012):

- As **emoções** são sensações que se caracterizam por ter menor duração, são mais intensas e são provocadas por algum estímulo. Por exemplo, se alguém for mal-educado com você, isso pode lhe provocar raiva. Esse sentimento, provavelmente, vai durar pouco tempo, talvez alguns segundos. As emoções são apresentadas por meio de expressões corporais e comportamentais, além de demonstrarem natureza específica como alegria, surpresa e tristeza.
- O **humor** é o background (experiência/pano de fundo), que se caracteriza por apresentar baixa intensidade e duração maior que as emoções, afetando o funcionamento da pessoa de forma global. O humor pode influenciar as emoções e vice-versa. Por exemplo, quando um colega critica você pela forma como falou com um cliente, você pode experienciar e/ou demonstrar uma emoção (raiva) em relação a um objeto específico (seu colega). No entanto, conforme a emoção vai diminuindo, você pode se sentir desanimado de maneira geral. Você começa a perceber que esse estado de desânimo não é o seu estado normal e começa a exagerar em relação a outros eventos. Esse estado se caracteriza como um humor. Outros exemplos de humor são: animação ou desânimo e estado de alta ou diminuição de energia.
- Já o **afeto** é mais conhecido como "sentimento", que é uma ampla extensão de sensações neurofisiológicas, que são experienciadas por meio das emoções e do humor, como exemplo, temos o prazer e desprazer, tensão e relaxamento, energia e cansaço.

Em outras palavras, o **afeto** é um termo genérico que abrange uma ampla gama de sentimentos que as pessoas experimentam. É um conceito guarda-chuva que engloba tanto emoções e humores. As **emoções** são sentimentos intensos que são direcionados a alguém ou alguma coisa. Já o **humor** é um "sentimento" menos intenso do que as emoções que influencia o funcionamento de forma geral.

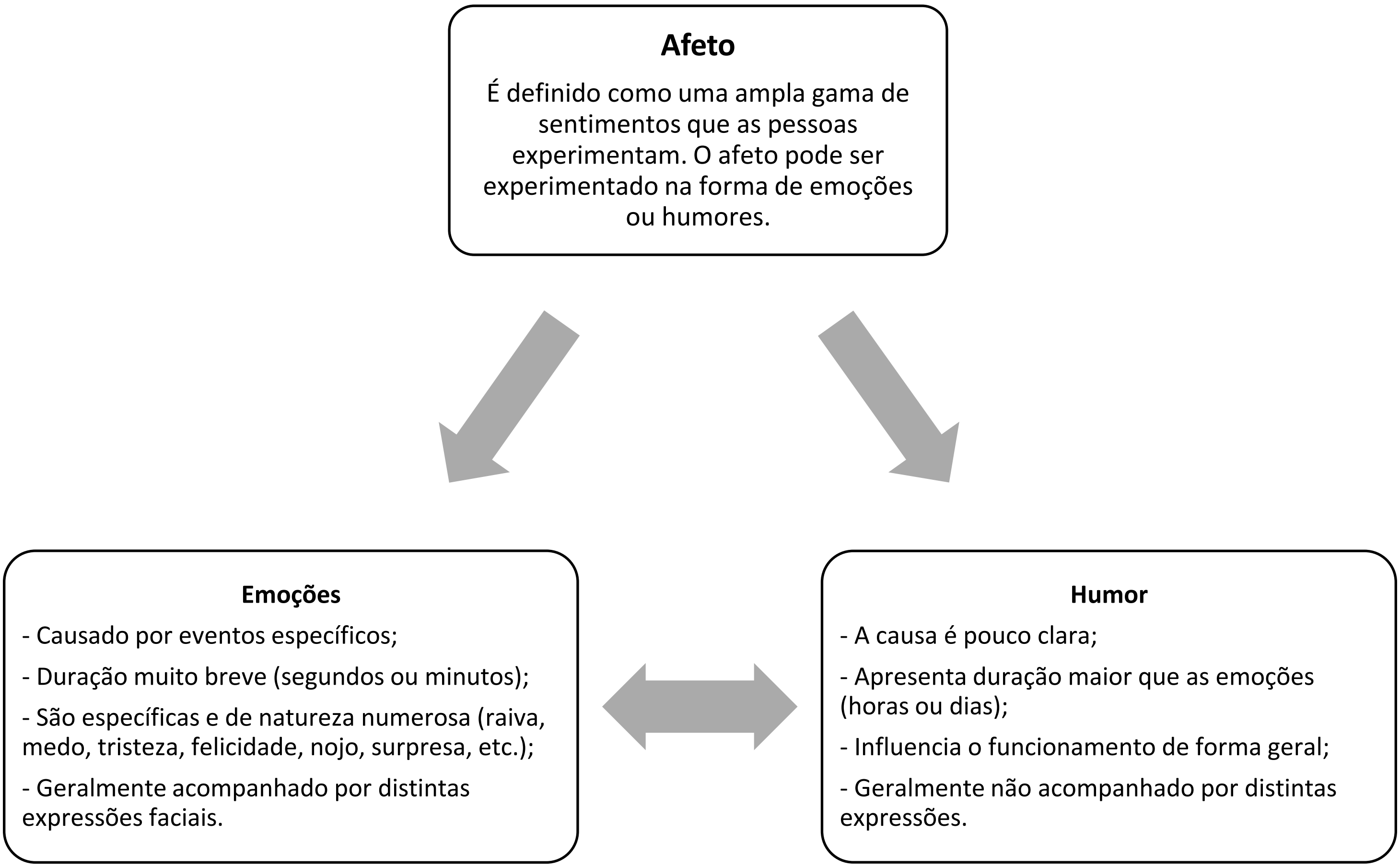

Figura 2. Diferenças entre afeto, emoções e humor. Figura traduzida e adaptada de Robbins & Judge (2012).

Quando as alterações do humor se tornam problemáticas?

O humor pode ser entendido como o estado de ânimo ou estado de espírito de uma pessoa, o qual costuma sofrer alterações no dia a dia. No entanto, para algumas pessoas, estas variações são mais importantes e persistentes, trazendo prejuízos para a vida do indivíduo. Esses quadros são chamados de Transtornos de Humor, e podem se manifestar através de sintomas depressivos (com ou sem ansiedade associada) ou sintomas maníacos (elevação do humor).

Quando a pessoa apresenta diminuições importantes e persistentes do humor, dizemos que seu humor está **deprimido**. Já quando a pessoa apresenta elevação do humor, dizemos que seu humor está **maníaco** ou **hipomaníaco**.

Esses sintomas (depressivos ou maníacos) podem gerar sintomas secundários como alterações no sono, aumento do peso e menor rendimento no trabalho. Quando não diagnosticado ou tratado corretamente, os Transtornos de Humor podem ocasionar prejuízos em diferentes aspectos da vida, como a perda do emprego, a baixa produtividade e a exposição a situações de risco. Consulte o seu médico ou terapeuta para ter mais detalhes.

INSTRUÇÕES DE PREENCHIMENTO DO LIFE CHART METHOD - SELF/PROSPECTIVE (LCM-S/P)

Reconhecendo seu humor

Os sintomas de mania, hipomania e depressão podem ser diferentes em cada pessoa. Esses sintomas podem durar algum período, podendo durar algumas semanas, mudar na mesma semana ou, até mesmo, no mesmo dia.

Para ajudar você a identificar o que sente e experiencia com seu humor, vamos listar alguns dos principais sintomas:

Sintomas Comuns de Mania/ Hipomania

- Aumento de energia;
- Sente-se agitado ou com o humor irritável;
- Autoestima inflada ou sentimento de grandiosidade;
- Diminuição da necessidade de sono;
- Sentir que tem falado mais que o habitual ou sentir uma pressão para continuar falando;
- Fuga de ideias ou pensamento acelerado;
- Facilmente distraído;
- Envolvimento excessivo em atividade prazerosa (gastos excessivos, atividade sexual, investimentos financeiros tolos).

Sintomas Comuns de Depressão

- Diminuição de energia;
- Humor deprimido, sentimento de vazio, tristeza;
- Diminuição do interesse ou prazer em atividades habituais;
- Dificuldade de concentração;
- Significativo ganho ou perda de peso;

- Mudança nos padrões de sono, incapacidade de dormir ou aumento dos períodos de sono;
- Sentimento de inutilidade ou culpa inadequada;
- Dificuldade em tomar decisões;
- Pensamentos recorrentes de morte ou suicídio.

Utilizando o LCM-S/P para Monitorar seu Humor

Este instrumento tem como objetivo auxiliar pessoas a monitorarem o seu próprio humor diariamente. Foi construído para fornecer um registro diário e mensal para você e o clínico que acompanha o seu tratamento.

É simples de usar, anotar o que vivenciou, como se sentiu, como se comportou, entre outros. É só seguir os passos abaixo para anotar as informações.

1. Medicamentos

Com a ajuda de algum profissional da saúde, liste os medicamentos que seu médico prescreveu nos espaços fornecidos, incluindo o nome do medicamento e a dosagem. A cada dia, indique o número exato de comprimidos que tomou de cada medicamento no espaço adequado. Liste todos os medicamentos que você ingeriu durante o dia incluindo psiquiátricos e não psiquiátricos.

2. Horas de Sono

Anote o número de horas que você dormiu na noite anterior.

3. Anote a gravidade dos seus episódios de humor

Nesta seção, indique como o seu humor afetou sua capacidade de atuar em seus papéis sociais e ocupacionais habituais (como em casa, no trabalho ou na escola). Para isso, preencha o espaço correspondente ao nível adequado de comprometimento funcional.

Instruções:

"Você, então, deve avaliar o quanto o seu humor tem afetado sua capacidade de funcionar durante o dia a dia em seus papéis habituais em casa, trabalho, escola e/ou com amigos. A intensidade ou a gravidade do humor é determinada com base no seu nível de comprometimento funcional (por exemplo: dificuldades com papéis sociais, educacionais e/ou

ocupacionais), que é afetado com a presença de sintomas depressivos ou maníacos/ hipomaníacos. Tire dois minutos, ao final do seu dia, e realize a avaliação do seu humor ao longo do dia. Preencha o círculo para realizar a marcação do seu humor."

Atenção:

- **Mania Disfórica:** o indivíduo experiencia sintomas de mania ou hipomania (como por exemplo: um aumento na energia, diminuição da necessidade de sono, aumento no número de atividades, de pensamentos ou de interações), associados com raiva, irritabilidade ou ansiedade. Portanto, quando o estado energizado é desagradável ou disfórico, por favor indique marcando um (✓), além de registrar a gravidade do estado de humor no quadro.
- Note que o comprometimento funcional, devido a outras doenças médicas, como uma gripe, uma perna quebrada, artrite, doenças cardíacas, entre outras, estes não devem ser levados em conta para avaliar a gravidade do episódio de humor.
- Se você acredita que não teve alterações no humor e comprometimento funcional ao longo do dia, você pode marcar na base de referência (neutro).
- Para alterações do humor que ocorreram no mesmo dia, faça um traço para cada alteração do humor entre o círculo mais elevado e o círculo mais baixo alcançados. Não é necessário a marcação dos círculos intermediários. Veja um exemplo na figura 3.

3.1 Níveis de hipomania e mania

Leve

- **Descrição:** no nível **leve** de hipomania, você pode sentir sintomas de forma suave, como: diminuição da necessidade do sono, aumento de energia, alguma irritabilidade ou euforia; ou um aumento na taxa de pensamento, de expressão ou de sociabilidade. No nível leve, esses sintomas não têm impacto negativo e podem até, inicialmente, melhorar a sua capacidade funcional.
- **Sintomas:** aumento de energia; aumento de atividades; mais sociável; entusiasmado; exuberante; irritável; falador; sente-se mais produtivo.
- **Comprometimento Funcional:** mínimo ou nenhum prejuízo funcional; continua a realizar bem as atividades no trabalho, escola e/ou em casa; o funcionamento pode até melhorar em algumas áreas.

Moderada Baixa

- **Descrição**: no nível **moderada baixa** de mania pode apresentar os sintomas do nível de mania leve, com uma intensidade um pouco maior e com sintomas adicionais. Você pode apresentar menor produtividade e dificuldades de manter o foco. Você pode começar a receber algum *feedback* de familiares, amigos ou colegas de trabalho, que seu comportamento está diferente do habitual.
- **Sintomas**: eufórico; irritável; intrusivo; comunicativo; disfuncional; insistente; diminuição da necessidade de sono; aumento de energia; pressionado; fuga de ideias; distraído; aumento de gastos; excesso de velocidade; aumento do interesse / atividade sexual; promíscuo; grandioso; pode ser imprudente.
- **Comprometimento Funcional:** dificuldade com atividades objetivas; sente-se produtivo, mas sem efetivamente ser (por exemplo, inicia muitos projetos sem conseguir terminar); ter problemas com o trabalho, escola e família; as pessoas começam a comentar sobre o seu comportamento.

Moderada Alta

- **Descrição**: no nível **moderada alta** de mania pode sentir sintomas muito significativos, como: muita diminuição da necessidade de sono (ou nem consegue dormir), um grande aumento no nível de energia, pode se sentir poderoso ou fora de controle, pode ter pensamentos e falas extremamente rápidos e receber muitos *feedbacks* que seu comportamento está diferente ou difícil. Outras pessoas podem expressar grandes preocupações com sua capacidade de cuidar de si mesmo ou dos outros, e podem parecer irritados ou frustrados com o seu comportamento.
- **Sintomas:** eufórico; irritável; intrusivo; muito comunicativo; disfuncional; insistente; diminuição da necessidade de sono; um grande aumento de energia; pressionado; fuga de ideias; muito distraído; um grande aumento de gastos; excesso de velocidade; um grande aumento do interesse/atividade sexual; promíscuo; grandioso; pode ser imprudente.
- **Comprometimento Funcional:** não consegue se concentrar; as pessoas ficam zangadas/frustradas com você; o seu julgamento está pobre; você tem grande dificuldade com atividades objetivas.

Grave

- **Descrição**: no nível **grave** do estado de humor maníaco há um aumento ainda maior dos sinais e sintomas descritos nos outros níveis, com muita insistência de familiares e amigos de que você precisa de atenção médica, que seu comportamento está fora de controle, ou eles podem levá-lo para um hospital. Há preocupações de que nem eles e nem você pode mantê-lo seguro por mais tempo. Se você estiver hospitalizado e com sintomas maníacos, marque este nível.
- **Sintomas:** sente pouca ou nenhuma necessidade de sono; sente-se fora de controle; explosivo; sente-se todo poderoso; invencível; bravo; potencialmente violento; sente-se com energia excessiva; imprudente; vê ou ouve coisas que não existem realmente.
- **Comprometimento Funcional:** necessita de supervisão constante; pediu para se afastar do trabalho ou escola; é incapaz de executar quaisquer atividades objetivas; comportamento ou decisões bizarras; seus familiares e amigos insistem que você precisa buscar alguma ajuda; você pode ter problemas com leis; necessidade de hospitalização psiquiátrica.

3.2 Níveis de depressão

Leve

- **Descrição**: o nível **leve** de depressão representa uma percepção subjetiva de sofrimento, um baixo-astral, alguns isolamentos sociais, mas continua com pouco ou nenhum comprometimento funcional.
- **Sintomas:** angústia subjetiva; humor triste leve; lentidão; "um pouco fora"; desinteresse leve; sono e apetite podem estar normais.
- **Comprometimento Funcional:** mínimo ou nenhum prejuízo; continua a exercer bem suas atividades no trabalho, escola e/ou em casa.

Moderada Baixa

- **Descrição**: no nível **moderada baixa** de depressão você pode perceber que suas habilidades funcionais, em alguns papéis habituais, são mais difíceis devido a sintomas de humor depressivo e requer tempo ou esforço extra para realizá-los.
- **Sintomas:** humor deprimido; sem esperança; falta de interesse; choroso; ansioso; irritável; concentração diminuída; diminuição de energia; diminuição da autoestima; sentimento de culpa; incapaz de aproveitar as coisas; nenhum interesse em coisas

prazerosas; ideação suicida; dificuldades para dormir; problemas com alimentação; retardo psicomotor e lentificação generalizada ou agitação psicomotora; diminuição do interesse/atividade sexual; bravo; socialmente retraído; isolamento social.

- **Comprometimento Funcional:** necessita de um esforço extra para executar atividades; ocasionalmente falta alguns dias de trabalho ou escola; prejuízo notável no trabalho, escola e/ou casa.

Moderada Alta

- **Descrição**: no nível **moderada alta** de depressão você pode perceber que suas habilidades funcionais são mais difíceis de serem realizadas, demandando de grande tempo extra ou grande esforço, pode apresentar também dificuldades em rotinas habituais.
- **Sintomas:** humor muito deprimido; sem esperança; falta de interesse; choroso; ansioso; irritável; concentração muito diminuída; energia muito baixa; diminuição da autoestima; sentimento de culpa; incapaz de aproveitar as coisas; nenhum interesse em coisas prazerosas; comportamento suicida; muita dificuldade para dormir; problemas com alimentação; retardo psicomotor e lentificação generalizada ou agitação psicomotora; diminuição do interesse/atividade sexual; bravo / irritável; socialmente retraído; isolamento social; pouca higiene.
- **Comprometimento Funcional:** muito esforço extra é necessário para executar atividades; prejuízo muito significativo no trabalho, na escola ou em casa; está faltando muitos dias no trabalho ou na escola.

Grave

- **Descrição:** no nível **grave** de depressão significa que você é incapaz de funcionar em qualquer uma de suas funções sociais e ocupacionais habituais, ou seja, é incapaz de sair da cama, ir para a escola ou trabalho, realizar qualquer uma das suas funções de rotina, exige muitos cuidados extras em casa, ou necessidade de ser hospitalizado.
- **Sintomas:** imobilizado; falta de autocuidado; má alimentação; baixa ingestão de líquidos; incapaz de se vestir; longos atrasos na fala ou mudo; muito agitado, andando de um lado para o outro; comportamento suicida; não consegue pensar ou lembrar; crenças falsas (delírios); distorções sensoriais (alucinações).

- **Comprometimento Funcional:** não está trabalhando ou indo a escola; não realiza atividades de casa; não consegue realizar qualquer atividade de rotina; está incapacitado em casa ou hospitalizado.

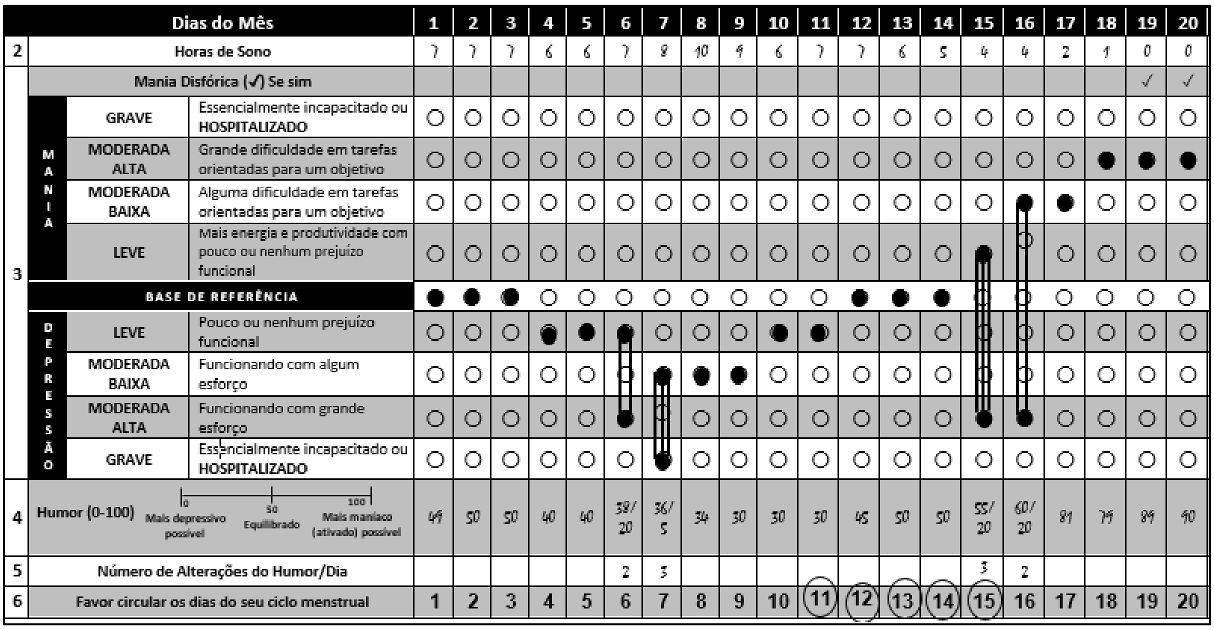

Figura 3. Exemplo de preenchimento da versão brasileira do *Life Chart Method - Self/Prospective* (LCM-S/P), construído pelos autores.

3.3 Sua Própria Lista de Verificação de Sintomas Típicos

Você também pode desenvolver sua própria lista de sintomas típicos associados a episódios depressivos e maníacos. Por exemplo, em algumas pessoas, o melhor marcador de hipomania pode ser o aumento de energia, enquanto, em outras, pode ser a diminuição da necessidade de sono ou maior sociabilidade, telefonemas ou gastos. Da mesma forma, para a depressão, algumas pessoas sentem-se lentas ou apáticas, enquanto outras se sentem agitadas. Algumas pessoas dormem mais, enquanto outras não conseguem dormir muito. Outras têm a impressão de que sua mente está vazia, enquanto outras são atormentadas por pensamentos depressivos.

Ter a própria lista de seus sintomas típicos pode servir como um sistema próprio de alerta precoce, que pode auxiliar você e seu clínico a estarem mais conscientes dos sinais de

suas alterações de humor. Dessa forma, você pode intervir desde cedo, antes que os sintomas fiquem fora de controle, perguntando ao seu clínico ou terapeuta como proceder.

4. Avalie seu Humor Geral

Avalie o seu humor de forma geral e escreva um número de 0 a 100, baseado na escala de humor demonstrada abaixo. Se você, no mesmo dia, tiver alterações de humor súbitas ou grandes, insira os valores de humor mais alto e mais baixo alcançados.

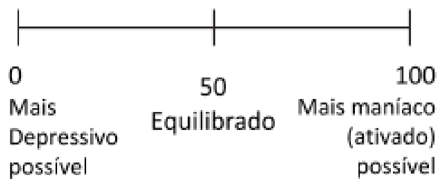

Figura 4. Métrica para avaliar o humor geral da versão brasileira do *Life Chart Method - Self/Prospective* (LCM-S/P), traduzida e adaptada da versão original do LCM-S/P (Leverich & Post, 2002).

5. Anote o Número de Alterações de Humor

Insira o número de mudanças de humor que ocorreram durante o dia (se houver). Ressalta-se que essas mudanças devem ser repentinas, distintas e significativas. Mudanças de humor podem ocorrer dentro do mesmo estado de humor ou entre estados de humor.

Atenção: Não conte a variação diurna gradual como mudança de humor; ao invés disso, avalie o nível mais severo de comprometimento funcional durante todo o dia.

6. Indique o seu Ciclo Menstrual (para pacientes do sexo feminino)

Circule os dias que represente o início e o fim do seu ciclo menstrual durante o mês.

7. Registre os Eventos de Vida Experienciados durante o Dia

Anote os eventos realmente relevantes que ocorreram ao longo do dia.

Anote o evento ocorrido e um valor sobre os efeitos ou desconfortos que esse evento lhe causou, seguindo a escala abaixo. O valor é baseado em algum impacto (positivo ou negativo) que essa experiência tenha lhe causado.

-4	-3	-2	-1	0	+1	+2	+3	+4
Extremamente Negativa				Neutro				Extremamente Positiva

Figura 5. Métrica para avaliar o impacto do evento de vida da versão brasileira do *Life Chart Method - Self/Prospective* (LCM-S/P), traduzida e adaptada da versão original do LCM-S/P (Leverich & Post, 2002).

8. Sintomas Comórbidos

Anote os sintomas adicionais que você pode experienciar no dia (como ansiedade, abuso de álcool, paranoia, dor de cabeça, entre outros). Se aplicável, continue a indicar a presença e a frequência desses sintomas nos dias subsequentes (por exemplo, número de ataques de pânico, número de bebidas alcoólicas, etc.).

Dia do Mês	Eventos da Vida	Impacto (-4 a +4)	Listar Sintomas Comórbidos
1			
2			
3	Briga com o melhor amigo	-3	
4			
5			
6			
7			
8			
9			Ataque de pânico
10			
11			
12			
13	Ganho de um promoção no trabalho	+3	
14			
15			
16			
17			Álcool: 5 drink/dia

Figura 6: Exemplo de preenchimento de evento de vida, nota sobre o impacto e sintomas comórbidos, construído pelos autores.

REFERÊNCIAS

Ekkekakis, P. (2013). *The measurement of affect, mood, and emotion: A guide for health-behavioral research.* Cambridge University Press.

Leverich, G. S. & Post, R. M. (2002). *The NIMH Life Chart Manual for Recurrent Affective Illness: The LCM - S/P (Self-Version/Prospective).* Bethesda, MD: NIMH.

Robbins, S. P. & Judge, T. A. (2012). *Organizational Behavior*. Bonston: Person.

Versão brasileira do Life Chart Method - Self/Prospective (LCM-S/P)

NIMH - Life Chart Method - Self/Prospective (LCM-S/P)

INFORMAÇÕES

Nome:____

____

Endereço:____

____

Número(s) de Telefone(s):

____

____

Número(s) de Telefone(s) para emergências:

____

____

Número(s) de Telefone(s) do seu médico e/ou terapeuta:

____

____

OUTROS NÚMEROS DE TELEFONES IMPORTANTES E OUTRAS ANOTAÇÕES:

____

____

____

____

____

____

____

____

____

____

____

HUMOR

O humor pode ser entendido como o estado de ânimo ou estado de espírito de uma pessoa, o qual costuma sofrer alterações no dia a dia. No entanto, para algumas pessoas, estas variações são mais importantes e persistentes, trazendo prejuízos para a vida do indivíduo. Esses quadros são chamados de Transtornos de Humor, e podem se manifestar através de sintomas depressivos (com ou sem ansiedade associada) ou sintomas maníacos (elevação do humor).

Lembre-se que o **humor** é diferente das emoções e vai muito além de estado de felicidade ou tristeza. Quando a pessoa apresenta diminuições importantes e persistentes do humor, dizemos que seu humor está **deprimido**. Já quando a pessoa apresenta elevações do humor importantes e persistentes, dizemos que seu humor está **maníaco** ou **hipomaníaco**.

Esses sintomas (depressivos ou maníacos) podem gerar sintomas secundários como alterações no sono, aumento do peso e menor rendimento no trabalho. Quando não diagnosticado ou tratado corretamente, os Transtornos de Humor podem ocasionar prejuízos em diferentes aspectos da vida, como a perda do emprego, a baixa produtividade e a exposição a situações de risco. Consulte o seu médico ou terapeuta para ter mais detalhes.

RECONHECENDO SEU HUMOR

Os sintomas de mania, hipomania e depressão podem ser diferentes em cada pessoa. Estes sintomas podem durar algum tempo, por vezes algumas semanas, podendo até mesmo mudar no mesmo dia.

Para ajudar você a identificar o que sente e experiencia com seu humor, vamos listar alguns dos principais sintomas:

SINTOMAS DE MANIA/HIPOMANIA

- Aumento de energia;
- Sente-se agitado ou com o humor irritável;
- Autoestima inflada ou sentimento de grandiosidade;
- Diminuição da necessidade de sono;
- Sentir que tem falado mais que o habitual ou sentir uma pressão para continuar falando;
- Fuga de ideias ou pensamento acelerado;
- Facilmente distraído;
- Envolvimento excessivo em atividade prazerosa (gastos excessivos, atividade sexual, investimentos financeiros tolos).

SINTOMAS DE DEPRESSÃO

- Diminuição de energia;
- Humor deprimido, sentimento de vazio, tristeza;
- Diminuição do interesse ou prazer em atividades habituais
- Dificuldade de concentração;
- Significativo ganho ou perda de peso;
- Mudança nos padrões de sono, incapacidade de dormir ou aumento dos períodos de sono;
- Sentimento de inutilidade ou culpa inadequada;
- Dificuldade em tomar decisões;
- Pensamentos recorrentes de suicídio ou morte.

UTILIZANDO O LCM-S/P PARA ANOTAR O SEU HUMOR

Este instrumento tem como objetivo auxiliar pessoas a monitorarem o seu próprio humor diariamente. Foi construído para fornecer um registro diário e mensal para você e para o clínico que acompanha o seu tratamento.

É simples de usar: é só anotar o que você vivenciou, como você se sentiu, como você se comportou, etc. Em seguida, basta seguir estes passos para registrar as informações.

1. Medicamentos

Com a ajuda de algum profissional da saúde, liste os medicamentos que seu médico prescreveu nos espaços fornecidos, incluindo o nome do medicamento e a dosagem. A cada dia, indique o número exato de comprimidos que tomou de cada medicamento no espaço adequado.

2. Horas de Sono

Anote o número de horas que você dormiu na noite anterior.

3. Anote a Gravidade dos seus Episódios de Humor

Nesta seção indique como o seu humor afetou sua capacidade de atuar em seus papéis sociais e ocupacionais habituais (como em casa, no trabalho ou na escola). Para isso, siga os passos:

1) Descubra qual é o seu polo de humor: depressivo, elevado (mania) ou estável (base de referência);
2) Preencha o círculo correspondente ao nível adequado de comprometimento funcional.

Atenção:

- Quando os sintomas de mania/hipomania são desagradáveis ou disfóricos, por favor indique marcando um visto (✓), além da gravidade do estado de humor no quadro.
- Note que quando houver comprometimento funcional devido a outras doenças médicas, como gripe, uma perna quebrada, artrite, doenças cardíacas, etc., estes não devem ser levados em conta para avaliar a gravidade do episódio de humor.
- Se você acredita que não teve alterações no humor e comprometimento funcional ao longo do dia, você pode marcar na base de referência (neutro).
- Para alterações do humor que ocorreram no mesmo dia, faça um traço para cada alteração do humor entre o círculo mais elevado e o círculo mais baixo alcançados. Não é necessário a marcação dos círculos intermediários.

Para mais informações sobre como anotar a severidade do seu humor, consulte o guia de preenchimento do LCM-S/P.

4. Humor (0-100)

Avalie o seu humor de forma geral e escreva um número de 0 a 100, baseado na escala de humor. Se no decorrer do mesmo dia haver alterações súbitas e significativas, insira os valores de humor mais altos e mais baixos alcançados.

5. Anote o Número de Alterações de Humor

Insira o número de mudanças de humor que ocorreram durante o dia (se houver). Ressalta-se que estas mudanças devem ser repentinas, distintas e significativas. Mudanças de humor podem ocorrer dentro do mesmo estado de humor ou entre estados de humor diferentes.

6. Indique o seu Ciclo Menstrual (para pacientes do sexo feminino)

Circule os dias que represente o início e o fim do seu ciclo menstrual durante o mês.

7. Registre os Eventos de Vida Experienciados Durante o Dia

Anote os eventos realmente relevantes que ocorreram ao longo do dia. Anote também, ao lado, um valor sobre os efeitos que esse evento lhe causou, seguindo a escala -4 a +4. O valor é baseado em algum impacto (positivo ou negativo) que esta experiência tenha lhe causado. Sendo -4, uma experiência extremamente negativa; 0 uma experiência neutra; e +4 uma experiência extremamente positiva.

-4	-3	-2	-1	0	+1	+2	+3	+4
Extremamente Negativa				Neutro				Extremamente Positiva

8. Sintomas Comórbidos

Anote os sintomas adicionais que você pode experienciar durante o dia (tais como ansiedade, abuso de álcool, paranoia, dor de cabeça, entre outros). Se aplicável, continue a indicar a presença e a frequência desses sintomas nos dias subsequentes (por exemplo, número de ataques de pânico, número de bebidas alcoólicas, etc.).

Trends Psychiatry Psychother. http://dx.doi.org/10.47626/2237-6089-2020-0140

NIMH - Life Chart Method - Self/Prospective (LCM-S/P)

Nome: Fulano **Mês:** Outubro **Ano:** 2020

	Nome do Medicamento	Dose por Comprimido	Unidade de Medida (mg, mcf, gm)	1	2	3	4	5	6	7	8	9	10	11	12	13	14	15	16	17	18	19	20	21	22	23	24	25	26	27	28	29	30	31
1 (Favor considerar todas as medicações utilizadas atualmente)				Inserir o NÚMERO TOTAL de comprimidos INGERIDOS por dia																														
	Lítio	300	mg	3	3	3	3	3	3	3	3	2	2	3	3	2	2	3	3	3	4	4	3	3	3	2	3	3	3	3	3	3	3	
	Carbamazepina	500	mg	3	3	3	3	3	4	4	4	4	4	4	4	4	4	3	0	0	0	0	0	0	0	0	0	0	2	2	3	3	3	
	Valproato de Sódio	200	mg	1	1	1	1	1	1	1	1	1	1	0	1	1	1	1	1	1	1	1	0	0	1	1	1	1	1	1	1	1	1	

	Dias do Mês		1	2	3	4	5	6	7	8	9	10	11	12	13	14	15	16	17	18	19	20	21	22	23	24	25	26	27	28	29	30	31
2	Horas de Sono		7	7	7	6	6	7	8	10	9	6	7	7	6	5	4	4	2	1	0	0	1	2	3	6	7	7	7	8	8	6	
3	Mania Disfórica (✓) Se sim																				✓	✓											
MANIA	GRAVE	Essencialmente incapacitado ou **HOSPITALIZADO**	○	○	○	○	○	○	○	○	○	○	○	○	○	○	○	○	○	○	○	○	○	○	○	○	○	○	○	○	○	○	○
MANIA	MODERADA ALTA	Grande dificuldade em tarefas orientadas para um objetivo	○	○	○	○	○	○	○	○	○	○	○	○	○	○	○	○	○	●	●	●	●	○	○	○	○	○	○	○	○	○	○
MANIA	MODERADA BAIXA	Alguma dificuldade em tarefas orientadas para um objetivo	○	○	○	○	○	○	○	○	○	○	○	○	○	○	○	●	●	○	○	○	○	●	○	○	○	○	○	○	○	○	○
MANIA	LEVE	Mais energia e produtividade com pouco ou nenhum prejuízo funcional	○	○	○	○	○	○	○	○	○	○	○	○	○	○	●	○	○	○	○	○	○	○	●	○	○	○	○	○	○	○	○
	BASE DE REFERÊNCIA		●	●	●	○	○	○	○	○	○	○	○	●	●	●	○	○	○	○	○	○	○	○	○	○	○	○	○	○	○	○	○
DEPRESSÃO	LEVE	Pouco ou nenhum prejuízo funcional	○	○	○	●	●	●	○	○	○	●	●	○	○	○	○	○	○	○	○	○	○	○	○	●	●	●	●	○	○	●	○
DEPRESSÃO	MODERADA BAIXA	Funcionando com algum esforço	○	○	○	○	○	○	●	●	●	○	○	○	○	○	○	○	○	○	○	○	○	○	○	○	○	○	○	●	●	○	○
DEPRESSÃO	MODERADA ALTA	Funcionando com grande esforço	○	○	○	○	○	●	○	○	○	○	○	○	○	○	●	●	○	○	○	○	○	○	○	○	○	○	○	○	○	○	○
DEPRESSÃO	GRAVE	Essencialmente incapacitado ou **HOSPITALIZADO**	○	○	○	○	○	○	●	○	○	○	○	○	○	○	○	○	○	○	○	○	○	○	○	○	○	○	○	○	○	○	○
4	Humor (0-100)	0 Mais depressivo possível — 50 Equilibrado — 100 Mais maníaco (ativado) possível	49	50	50	40	40	38/20	36/5	34	30	30	30	45	50	50	55/20	60/20	81	79	89	90	90	80	55	45	45	40	39	35	35	41	
5	Número de Alterações do Humor/Dia							2	3								3	2															
6	Favor circular os dias do seu ciclo menstrual		1	2	3	4	5	6	7	8	9	10	(11)	(12)	(13)	(14)	(15)	16	17	18	19	20	21	22	23	24	25	26	27	28	29	30	31

EXEMPLO

Trends Psychiatry Psychother. http://dx.doi.org/10.47626/2237-6089-2020-0140

NIMH - Life Chart Method - Self/Prospective (LCM-S/P)

Mês: Outubro

Ano: 2020

	7		8
**Dia do Mês**	**Eventos da Vida**	**Impacto (-4 a +4)**	**Listar Sintomas Comórbidos**
1			
2			
3	Briga com o melhor amigo	-3	
4			
5			
6			
7			
8			
9			Ataque de pânico
10			
11			
12			
13	Ganho de uma promoção no trabalho	+3	
14			
15			
16			
17			Álcool: 8 drink/dia
18			
19			
20			
21			
22			
23			
24			
25			Tonturas
26			
27			
28			
29			
30			
31			

EXEMPLO

-4 Extremamente Negativa — **0** Neutro — **+4** Extremamente Positiva

Trends Psychiatry Psychother. http://dx.doi.org/10.47626/2237-6089-2020-0140

NIMH - Life Chart Method - Self/Prospective (LCM-S/P)

Nome: ______________________________ **Mês:** ______________________________ Ano: ______________________________

	Nome do Medicamento	Dose por Comprimido	Unidade de Medida (mg, mcf, gm)	Dias do Mês																														
				1	2	3	4	5	6	7	8	9	10	11	12	13	14	15	16	17	18	19	20	21	22	23	24	25	26	27	28	29	30	31
1 Favor considerar todas as medicações utilizadas atualmente				Inserir o NÚMERO TOTAL de comprimidos INGERIDOS por dia																														
	Lítio																																	
	Carbamazepina																																	
	Valproato de Sódio																																	

		Dias do Mês		1	2	3	4	5	6	7	8	9	10	11	12	13	14	15	16	17	18	19	20	21	22	23	24	25	26	27	28	29	30	31
2		Horas de Sono																																
3		Mania Disfórica (√) Se sim																																
	MANIA	GRAVE	Essencialmente incapacitado ou **HOSPITALIZADO**	○	○	○	○	○	○	○	○	○	○	○	○	○	○	○	○	○	○	○	○	○	○	○	○	○	○	○	○	○	○	○
	MANIA	MODERADA ALTA	Grande dificuldade em tarefas orientadas para um objetivo	○	○	○	○	○	○	○	○	○	○	○	○	○	○	○	○	○	○	○	○	○	○	○	○	○	○	○	○	○	○	○
	MANIA	MODERADA BAIXA	Alguma dificuldade em tarefas orientadas para um objetivo	○	○	○	○	○	○	○	○	○	○	○	○	○	○	○	○	○	○	○	○	○	○	○	○	○	○	○	○	○	○	○
	MANIA	LEVE	Mais energia e produtividade com pouco ou nenhum prejuízo funcional	○	○	○	○	○	○	○	○	○	○	○	○	○	○	○	○	○	○	○	○	○	○	○	○	○	○	○	○	○	○	○
	BASE DE REFERÊNCIA			○	○	○	○	○	○	○	○	○	○	○	○	○	○	○	○	○	○	○	○	○	○	○	○	○	○	○	○	○	○	○
	DEPRESSÃO	LEVE	Pouco ou nenhum prejuízo funcional	○	○	○	○	○	○	○	○	○	○	○	○	○	○	○	○	○	○	○	○	○	○	○	○	○	○	○	○	○	○	○
	DEPRESSÃO	MODERADA BAIXA	Funcionando com algum esforço	○	○	○	○	○	○	○	○	○	○	○	○	○	○	○	○	○	○	○	○	○	○	○	○	○	○	○	○	○	○	○
	DEPRESSÃO	MODERADA ALTA	Funcionando com grande esforço	○	○	○	○	○	○	○	○	○	○	○	○	○	○	○	○	○	○	○	○	○	○	○	○	○	○	○	○	○	○	○
	DEPRESSÃO	GRAVE	Essencialmente incapacitado ou **HOSPITALIZADO**	○	○	○	○	○	○	○	○	○	○	○	○	○	○	○	○	○	○	○	○	○	○	○	○	○	○	○	○	○	○	○
4	Humor (0-100)	0 Mais depressivo possível; 50 Equilibrado; 100 Mais maníaco (ativado) possível																																
5		Número de Alterações do Humor/Dia																																
6		Favor circular os dias do seu ciclo menstrual		1	2	3	4	5	6	7	8	9	10	11	12	13	14	15	16	17	18	19	20	21	22	23	24	25	26	27	28	29	30	31

Trends Psychiatry Psychother. http://dx.doi.org/10.47626/2237-6089-2020-0140

NIMH - Life Chart Method - Self/Prospective (LCM-S/P)

Mês: ______________________________

Ano: ______________________________

	7		8
**Dia do Mês**	**Eventos da Vida**	**Impacto (-4 a +4)**	**Listar Sintomas Comórbidos**
1			
2			
3			
4			
5			
6			
7			
8			
9			
10			
11			
12			
13			
14			
15			
16			
17			
18			
19			
20			
21			
22			
23			
24			
25			
26			
27			
28			
29			
30			
31			

-4 Extremamente Negativa — **0** Neutro — **+4** Extremamente Positiva

Trends Psychiatry Psychother. http://dx.doi.org/10.47626/2237-6089-2020-0140
